# Strain‐specific differential expression of astrocytes and microglia in the mouse hippocampus

**DOI:** 10.1002/brb3.961

**Published:** 2018-03-22

**Authors:** Jong Whi Kim, Sung Min Nam, Dae Young Yoo, Hyo Young Jung, In Koo Hwang, Je Kyung Seong, Yeo Sung Yoon

**Affiliations:** ^1^ Department of Anatomy and Cell Biology College of Veterinary Medicine and Research Institute for Veterinary Science Seoul National University Seoul Korea; ^2^ Department of Anatomy College of Veterinary Medicine Konkuk University Seoul Korea; ^3^ Department of Anatomy College of Medicine Soonchunhyang University Cheonan‐Si Korea; ^4^ KMPC (Korea Mouse Phenotyping Center) Seoul National University Seoul Korea

**Keywords:** astrocyte, genetic background, hippocampus, microglia, mouse strain

## Abstract

**Introduction:**

Genetic background influences neurotransmitter expression and function of the hippocampus. Genetic background influences the phenotype of the hippocampus, but expression of neuroglia in hippocampus has not been well established dependent on various mouse strains.

**Objectives:**

In this study, we investigated the effects of genetic background on cell population of astrocytes and microglia in eight widely used inbred strains (C57BL/6J, A/J, BALB/c, C3H/HeJ, FVB, 129/SvJ, DBA/1, and DBA/2) and one outbred strain (ICR).

**Methods:**

In all mouse strains, glial fibrillary acidic protein (GFAP)‐immunoreactive astrocytes and ionized calcium‐binding adaptor molecule 1 (Iba‐1)‐immunoreactive microglia were found in almost all layers of hippocampal CA1‐4 regions and the dentate gyrus.

**Results:**

We observed significant differences in the number of astrocytes and microglia. In the CA1 and CA3 regions, the number of GFAP‐immunoreactive astrocytes was highest in the C3H/HeJ strain, and lowest in the 129/SvJ and FVB strains. In the polymorphic layer of the dentate gyrus, the number was highest in the DBA/1 strain and lowest in the 129/SvJ strain. Among the nine mouse strains, the number of Iba‐1‐immunoreactive microglia was highest in the CA1 and CA3 regions in the ICR and in the dentate gyrus of the C57BL/6 strain. The CA1 region of the FVB strain and the CA3 region and dentate gyrus of DBA/2 had the lowest number of Iba‐1‐immunoreactive microglia.

**Conclusion:**

These results suggest that the numbers of astrocytes and microglia differ depending on the mouse strain and these differences may be related to strain‐dependent function of astrocytes.

## 1 INTRODUCTION

In the central nervous system (CNS), neuroglial cells consist mainly of astrocytes, microglia, and oligodendrocytes. Neuroglial cells serve as the innate immune system of the CNS in addition to maintaining structural support for the CNS. Each astrocyte and microglia has characteristic functions and cell morphologies. Astrocytes perform various functions in a normal healthy brain such as formation of the blood–brain barrier (BBB) (Abbott, Ronnback, & Hansson, 2006), production of proinflammatory cytokines, regulation of brain blood flow, regulation of synaptic interaction, uptake of neurotransmitters, and ion homeostasis (Allen & Barres, 2009; Perez‐Alvarez & Araque, 2013). Astrocytes outnumber neurons and form the highest population of neuroglial cells in the CNS. Microglial cells are the resident mononuclear phagocytes of the brain. They regulate innate immune responses and serve as the defense mechanism of the CNS. Microglia in the mouse brain constitute up to 20% of the total glial cell population (Lawson, Perry, & Gordon, 1992) and are divided into two subtypes, (1) resident microglial cells found in the CNS parenchyma and present from the embryonic stage and (2) replaced microglia from migrated circulating monocytes and other bone marrow progenitors (Kennedy & Abkowitz, 1997; Vallieres & Sawchenko, 2003). Astrocytes and microglia are normally present in the hippocampus where they act as immune cells and regulate the microenvironmental cell niche.

Accumulating evidence demonstrates that astrocytes and microglia play a complex role in the integration of mature neurons (Gomes, Spohr, Martinez, & Moura Neto, 2001). Resting state microglial cells, as well as the interaction between microglia and astrocytes, play a key role in maintaining the CNS and recent studies have aimed to understand the relationship between neuroglia, mature neurons, and neural stem cells (NSCs) (Gomes et al., 2001). In a neurogenic niche, astrocytes and microglia support and interact with NSCs via a cell‐to‐cell mechanism. Contact between astrocytes and NSCs regulates neuronal differentiation of NSCs in the subgranular zone of the dentate gyrus through instructive juxtacrine ephrin‐B signaling (Ashton et al., 2012) Microglia can also modulate hippocampal neural precursor activity through the CX3CL1–CX3CR1 axis (Vukovic, Colditz, Blackmore, Ruitenberg, & Bartlett, 2012). The above results demonstrate that astrocytes, microglia, and NSCs interact in the dentate gyrus, and the role of astrocytes and microglia is not limited to their immune response functions.

Under normal healthy conditions, microglia exists in resting state‐shaped ramified morphology and show weak expression of antigen markers. In addition, astrocytes have a thread‐like morphology with a small cytoplasm and perform normal adaptive functions. During acute injury or ischemic states, astrocytes react and undergo cellular swelling, known as astrogliosis or astrocytosis, evidenced by a hypertrophied cytoplasm with increased expression of the glial fibrillary acidic protein (GFAP) (Li, Chopp, Zhang, & Zhang, 1995; Li et al., 2008). Under similar conditions of acute injury or ischemia, microglia show morphological changes such as cell body enlargement and shortening of cellular processes and migrate into injury sites with the activation of a phagocytosis phase (Colton & Wilcock, 2010). Chronic inflammation induced by lipopolysaccharide (LPS) infusion increases the population of both astrocytes and microglia in the hippocampus of rats (Hauss‐Wegrzyniak, Dobrzanski, Stoehr, & Wenk, 1998). Research on the activation of astrocytes and microglia under inflammatory conditions has concentrated on neurodegeneration with aging (Long et al., 1998) and in neurological disease, for example in Alzheimer's disease (AD) (McGeer & McGeer, 1995), stroke (Hu et al., 2012), and traumatic brain damage (Kumar et al., 2013). Most research in this area has investigated a change in population size and activation and morphological change in neuroglia within the hippocampus. Inflammatory reactions subsequently activate astrocytes and microglial cells reactivity, with upregulation in the level of circulating cytokines (Long et al., 1998). However, under normal healthy conditions, astrocytes and microglial cell populations maintain a normal status and sustain inactive morphological characteristics. In this study, our aim was to define mouse strain‐dependent variation in astrocytes and microglial populations under normal young‐age conditions.

Genetic background influences the phenotype of many organs including the brain, especially the hippocampus, and is dependent on function and protein expression in various mouse strains. The population of hippocampal NSCs differs among mouse strains, and genetic influence on hippocampal neurogenesis has been investigated in C57BL/6, BALB/c, ICR, and 129/SvJ (Kempermann, Kuhn, & Gage, 1997). In behavioral tests, inbred mice showed different scores in various behavioral paradigms such as the rotarod test, open field activity, and habituation, as well as contextual and cued fear conditioning (Bothe, Bolivar, Vedder, & Geistfeld, 2004; Crawley et al., 1997). Stereological analysis showed that the total number of astrocytes and microglial cells in each hippocampal region, DG and CA1, differs depending on age in C57BL/6J mice (Long et al., 1998). Inherent hippocampal function like long‐term potentiation and synaptic plasticity‐dependent memory was processed in dorsal hippocampus (Bannerman et al., 2014). Astrocytes and microglia are abundant in the hippocampal region, including in the CA1‐3 region and dentate gyrus.

In the last decade, accumulating evidence has been compiled in the Mouse Phenome Database about the characteristics of inbred mouse strains (Grubb, Churchill, & Bogue, 2004). For example, C57BL/6J mice have been commonly used in the field of neuroscience; however, this strain has rarely been used in models of cerebral amyloidosis compared with other mouse strains such as the 129S1/ SvImJ (Lehman et al., 2003), DBA/2J (Lehman et al., 2003), A/J (Sebastiani et al., 2006), SJL (Carlson et al., 1997), and Swiss Webster (Carter, Pedrini, Ghiso, Ehrlich, & Gandy, 2006). However, a few studies have employed comprehensive analysis to compare the location and number of astrocytes and microglia in the dorsal part of hippocampus of various strains of mice. In this study, we compared the normal distribution and stereologically analyzed the number of astrocytes and microglia in the dorsal hippocampus of the eight most widely used inbred strains (C57BL/6J, A/J, BALB/c, C3H/HeJ, FVB, 129/SvJ, DBA/1, and DBA/2) as well as one outbred (ICR) strain of mice.

## 2 MATERIALS AND METHODS

### 2.1 Experimental animals

Six‐week‐old male C57BL/6J, A/J, BALB/c, C3H/HeJ, FVB, 129/SvJ, DBA/1, DBA/2, and ICR mice were purchased from Japan SLC, Inc. (Shizuoka, Japan). The animals were housed in a specific pathogen‐free animal facility at 23°C with 60% humidity with a 12 hr/12 hr light/dark cycle and had ad libitum access to food and tap water. The handling and care of the animals conformed to guidelines established in compliance with current international laws and policies (NIH Guide for the Care and Use of Laboratory Animals, NIH Publication No. 85‐23, 1985, revised 2011) and were approved by the Institutional Animal Care and Use Committee (IACUC) of Seoul National University (SNU‐120913‐1‐2). All experiments were conducted with an effort to minimize the number of animals used and the suffering caused by the procedures used in the study. All mice of each strain (*n *=* *5 per strain) were sacrificed at 12 weeks and processed for tissue preparation.

### 2.2 Tissue preparation

Animals (*n *=* *5 in each group) were anesthetized using an intraperitoneal injection of 1.5 g/kg urethane (Sigma‐Aldrich, St. Louis, MO, USA) and perfused transcardially with 0.1 m phosphate‐buffered saline (PBS, pH 7.4) followed by 4% paraformaldehyde in 0.1 m PBS. The brains were then dissected and postfixed in the same fixative for 12 h. The brain tissues were cryoprotected using infiltration with 30% sucrose overnight. Thirty‐micrometer thick brain sections were serially obtained in the coronal plane using a cryostat (Leica, Wetzlar, Germany) and collected in six‐well plates containing PBS at −20°C for further processing.

### 2.3 Immunohistochemistry

To obtain accurate data for immunohistochemistry, the free‐floating sections from all animals were processed carefully under the same conditions. For each animal, tissue sections were selected between 1.46 and 2.46 mm posterior to the bregma by referring to the mouse atlas by Franklin and Paxinos (Franklin & Paxinos, 2013). Ten sections, 90 μm apart, were sequentially treated with 0.3% hydrogen peroxide (H_2_O_2_) in PBS for 30 min and 10% normal goat serum (Vector Laboratories, Burlingame, CA, USA) in 0.05 m PBS for 30 min. They were then incubated with a rabbit anti‐glial fibrillary acidic protein (GFAP, 1:1,000; Abcam, Cambridge, UK) or rabbit anti‐ionized calcium‐binding adapter molecule 1 (Iba‐1, 1:50; Wako, Osaka, Japan) overnight at 25°C and subsequently treated with either a biotinylated goat anti‐rabbit IgG, or a streptavidin‐peroxidase complex (1:200, Vector Laboratories). Sections were visualized by treating with 3,3′‐diaminobenzidine tetrachloride (Sigma) in 0.1 m Tris‐HCl buffer (pH 7.2) and dehydrated and mounted in Canada balsam (Kanto Chemical, Tokyo, Japan) onto gelatin‐coated slides.

### 2.4 Stereology

Two observers performed the analysis of the number of GFAP‐immunoreactive astrocytes and Iba‐1‐immunoreactive microglia for each experiment. In order to ensure objectivity in blind conditions, each observer carried out the measures under the same conditions. Sections were cut at 30‐μm to allow for a 20‐μm optical dissector within each section after dehydration and dehydration. Digital images of the mid‐point of the stratum radiatum in the hippocampal CA1 and CA3 region were captured with a Pannoramic Scan II with motorized Z‐stack (3DHISTECH, Budapest, Hungary). We counted the number of GFAP‐immunoreactive astrocytes and Iba‐1‐immunoreactive microglia based on the stereology using StereoInvestigator software (MBF, VT, USA). The counting frame width (X) and height (Y) was 0.25 mm producing a counting frame area (XY) of 0.0625 mm^2^. The dissector height (Z) was 20‐μm creating a dissector volume (XYZ) of 0.00125 mm^3^. Polymorphic layer of dentate gyrus area fraction was measured using StereoInvestigator (MBF, VT, USA) in each slide of a sample (because every polymorphic layer of dentate gyrus does not have the same area fraction) and counted for each section area with the dissector height (Z) was 20‐μm creating a dissector volume (XYZ) and calibrated a dissector volume (XYZ) of 0.00125 mm^3^. (200× primary magnification).

### 2.5 Statistics

Statistical analysis was performed using SPSS V.20.1 (IBM Corporation, Armonk, NY, USA). Experimental groups were compared using one‐way analysis of variance (anova), followed by a Tukey's honest significant difference post hoc analysis.

## 3 RESULTS

### 3.1 Localization of GFAP‐immunoreactive astrocytes in the hippocampal CA1 region

**Figure 1:**
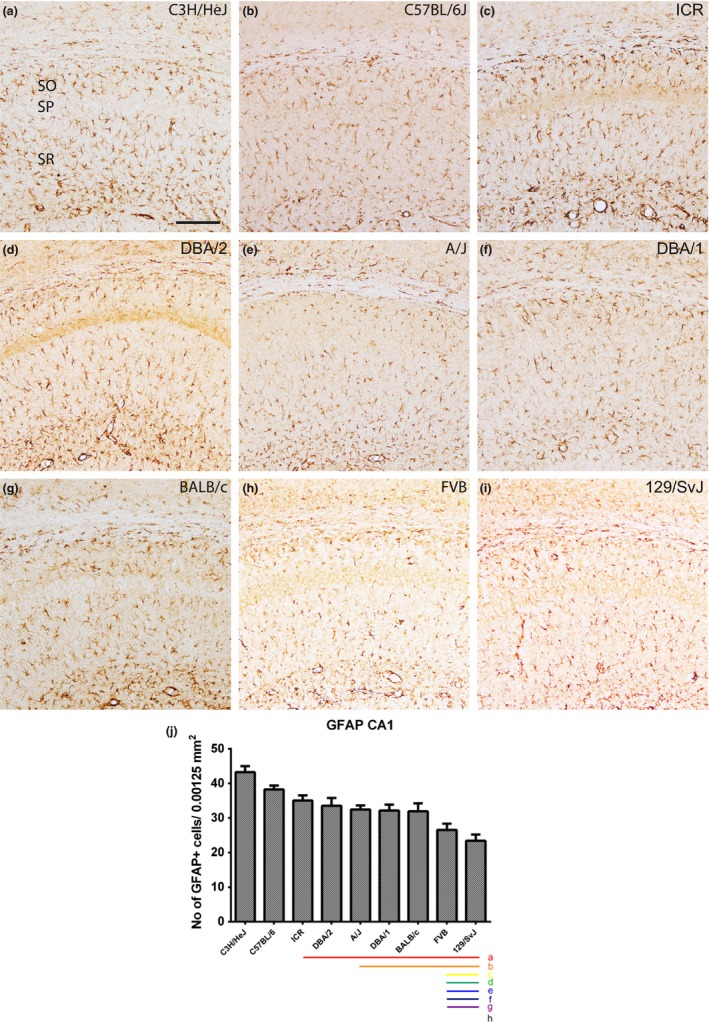
GFAP immunostaining in the CA1 region and quantitative analysis of the number of GFAP‐immunoreactive astrocytes in the stratum radiatum (SR) of the CA1 region of the hippocampus. GFAP‐labeled astrocytes are detected in the stratum oriens (SO) and SR of the CA1 region in all nine mouse strains; however, GFAP‐immunoreactive astrocytes show differences dependent on the mouse strain (a–i). SP, stratum pyramidal astrocytes. Scale bar = 100 μm. The population of GFAP‐immunoreactive astrocytes counted in nine mouse strains (j). Significant differences between the mouse strains are highlighted under the graph (j). “a” and a red bar indicate a significant difference compared to C3H/HeJ, “b” and orange bar, C57BL/6; “c” and yellow bar, ICR; “d” and green bar, DBA/2; “e” and blue bar, A/J; “f” and navy bar, DBA/1; “g” and purple bar, BALB/c; “h” and black bar, FVB;* p* < .05. Data are represented as mean ± *SEM*

In all nine mouse strains, GFAP‐immunoreactive astrocytes were found in the stratum radiatum, stratum oriens, and stratum lacunosum‐moleculare of the CA1 region. GFAP‐immunoreactive astrocytes showed similar morphologies in the hippocampal CA1 region of all mouse strains, but the numbers of GFAP‐immunoreactive astrocytes were significantly different between the strains (Figure 1a–i). The number of GFAP‐immunoreactive astrocytes in the stratum radiatum of the CA1 region was highest in C3H/HeJ strain (43.24 ± 1.73) and lowest in the 129/SvJ strain (23.38 ± 1.81) (Figure 1j). C57BL/6 strain also showed a relatively high number of GFAP‐immunoreactive cells, while ICR DBA/2, A/J, DBA/1, and Balb/c showed a relatively intermediate number, and FVB and 129/SvJ showed a relatively lower number of GFAP‐immunoreactive cells (Figure 1j). Statistical analysis was performed between mouse strains and showed in Figure 1j.

### 3.2 Localization of GFAP‐immunoreactive astrocytes in the CA3 region

**Figure 2:**
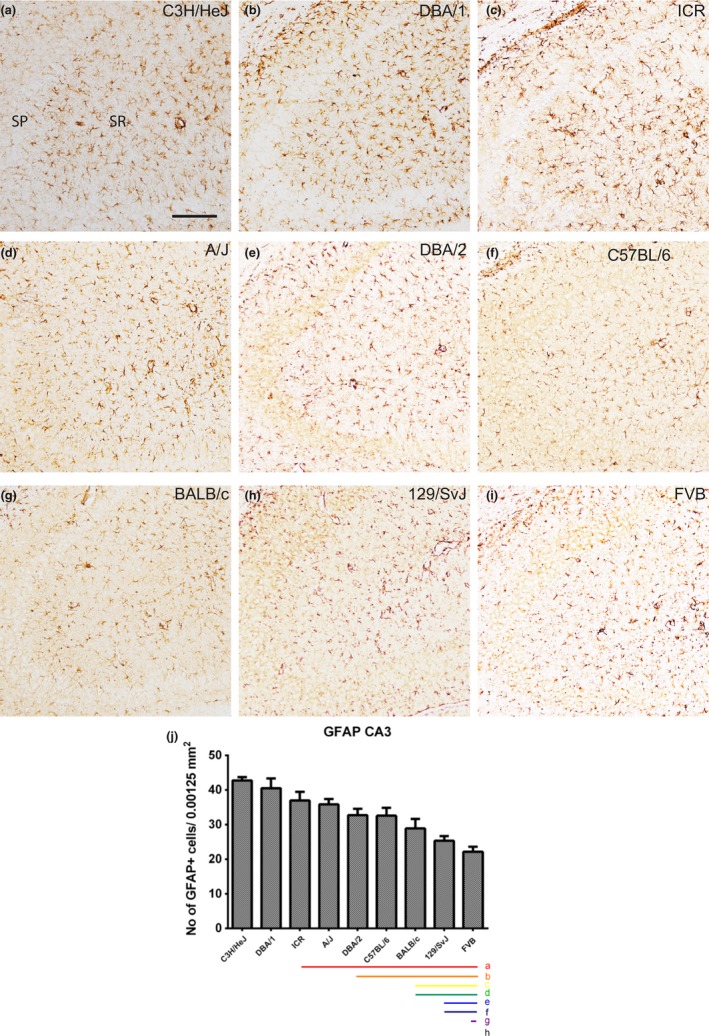
GFAP immunostaining in the CA3 region and quantitative analysis of the number of GFAP‐immunoreactive astrocytes in the stratum radiatum (SR) of the CA3 region of the hippocampus. GFAP‐labeled astrocytes are detected in the stratum oriens and SR of the CA3 region in all nine mouse strains; however, GFAP‐immunoreactive astrocytes show differences dependent on the mouse strain (a–i). SP, stratum pyramidal astrocytes. Scale bar = 100 μm. The population of GFAP‐immunoreactive astrocytes counted in nine mouse strains (j). Significant differences between the mouse strains are highlighted under the graph (j). “a” and a red bar indicate a significant difference compared to C3H/HeJ; “b” and orange bar, DBA/1; “c” and yellow bar ICR, “d” and green bar, A/J; “e” and blue bar, DBA/2; “f” and navy bar, C57BL/6; “g” and purple bar, BALB/c; “h” and black bar, 129/SvJ; *p* < .05. Data are represented as mean ± *SEM*

In the hippocampal CA3 region, we observed significant differences in the number of GFAP‐immunoreactive astrocytes among the nine mouse strains (Figure 2a–i). Quantitative analysis of GFAP‐immunoreactive astrocytes demonstrated that the C3H/HeJ (42.73 ± 0.96) had the highest number, whereas the FVB (22.13 ± 1.45) strain (Figure 2j) had the lowest numbers of GFAP‐immunoreactive astrocytes in the CA3 hippocampal region. C3H/HeJ showed a relatively high number of GFAP‐immunoreactive astrocytes, with strains DBA/1, ICR, A/J, DBA/2, C57BL/6, BALB/c, 129/SvJ, and FVB progressively showing a decrease in the number of GFAP‐immunoreactive astrocytes (Figure 2j). Statistical analysis was also performed between mouse strains and showed in Figure 2j.

### 3.3 Localization of GFAP‐immunoreactive astrocytes in the polymorphic layer of the dentate gyrus

**Figure 3:**
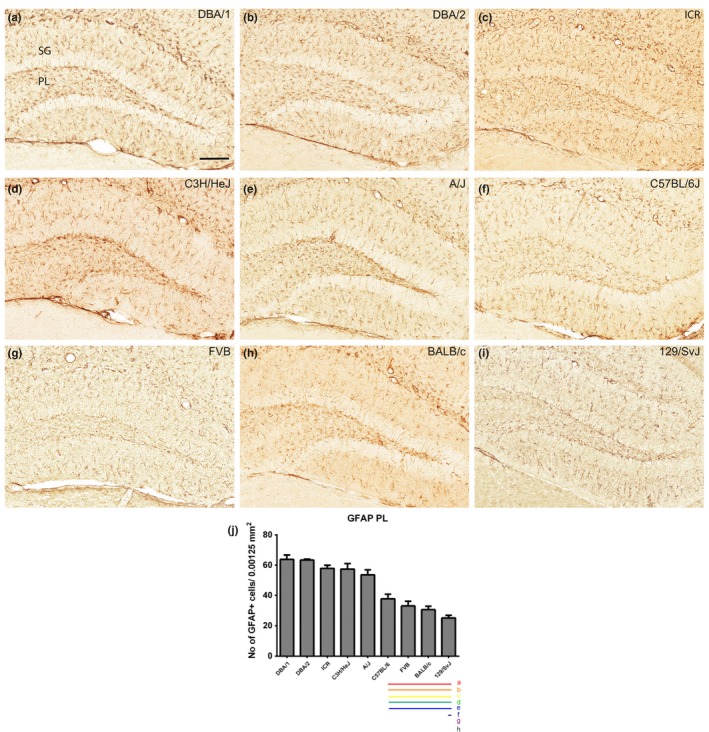
GFAP immunostaining in the dentate gyrus and quantitative analysis of the number of GFAP‐immunoreactive astrocytes in the polymorphic layer (PL) of the dentate gyrus. GFAP‐labeled astrocytes are detected in the molecular layer (ML) and PL of the dentate gyrus in all nine mouse strains; however, GFAP‐immunoreactive astrocytes show differences dependent on mouse strain (a–i). GCL, granule cell layer. Scale bar = 100 μm. The population of GFAP‐immunoreactive astrocytes counted in nine mouse strain (j). Significant differences between the mouse strains are highlighted under the graph (j). “a” and a red bar indicate a significant difference compared to DBA/1; “b” and orange bar, DBA/2; “c” and yellow bar, ICR; “d” and green bar, C3H/HeJ; “e” and blue bar, A/J; “f” and navy bar, C57BL/6; “g” and purple bar, FVB; “h” and black bar, BALB/c); *p* < .05. Data are represented as mean ± *SEM*

In the dentate gyrus, GFAP‐immunoreactivity was found in the polymorphic layer of the dentate gyrus in all nine mouse strains. In this study, we analyzed the GFAP‐immunoreactive astrocyte number in the polymorphic layer of the dentate gyrus and observed significant differences in the number of GFAP‐immunoreactive astrocytes among the nine mice strains (Figure 3a–i). The number of GFAP‐immunoreactive astrocytes in the polymorphic layer of the dentate gyrus was highest in the DBA/1 (63.84 ± 2.87) and lowest in the 129/SvJ (25.15 ± 1.74) strain (Figure 3j). DBA/1, DBA/2, ICR, C3H/HeJ, and A/J strains showed relatively high numbers of GFAP‐immunoreactive astrocytes, whereas C57BL/6, FVB, Balb/c and 129/SvJ strains showed a relatively low number of GFAP‐immunoreactive astrocytes (Figure 3j). Statistical analysis was also performed between mouse strains and showed in Figure 3j.

### 3.4 Localization of Iba‐1‐immunoreactive microglia in the CA1 region

**Figure 4:**
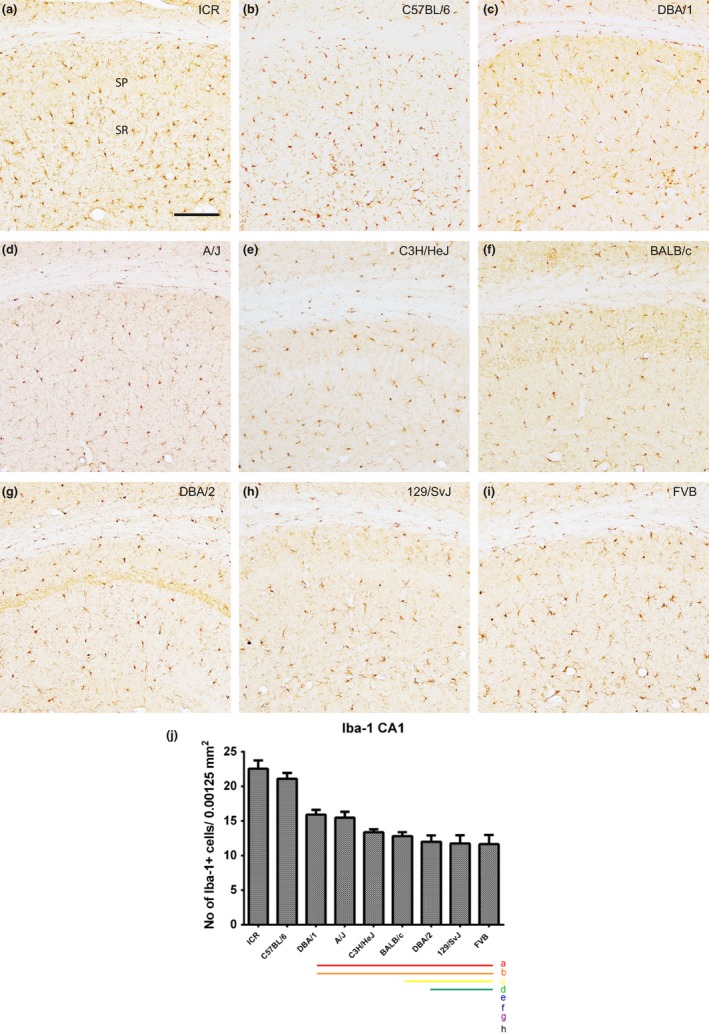
Iba‐1 immunostaining in the CA1 region and quantitative analysis of the number of Iba‐1‐immunoreactive microglia in the stratum radiatum (SR) of the CA1 region of the hippocampus. Iba‐1‐immunoreactive microglia are observed in the stratum oriens (SO) and SR of the CA1 region in all nine mouse strains; however, Iba‐1‐immunoreactive microglia show differences dependent on mouse strain (a–i). SP, stratum pyramidal microglia. Scale bar = 100 μm. The population of Iba‐1‐immunoreactive microglia counted in nine mouse strain (j). Significant differences between the mouse strains are highlighted under the graph (j). “a” and a red bar indicate a significant difference compared to ICR; “b” and orange bar, C57BL/6; “c” and yellow bar, DBA/1; “d” and green bar, A/J; “e” and blue bar, C3H/HeJ; “f” and navy bar, BALB/c; “g” and purple bar, DBA/2; “h” and black bar, 129/SvJ; *p* < .05. Data are represented as mean ± *SEM*

Iba‐1‐immunoreactive microglia were detected in both the stratum oriens and radiatum in the CA1 region, and the number of Iba‐1‐immunoreactive microglia in the stratum radiatum was analyzed. We observed significant differences in the number of Iba‐1‐immunoreactive microglia with respect to mouse strain (Figure 4a–i). The number of Iba‐1‐immunoreactive microglial cells was highest in the ICR (26.93 ± 0.65) and lowest in the FVB (12.26 ± 1.32) strain (Figure 4j). ICR and C57BL/6 had a relatively high number of Iba‐1‐immunoreactive cells, while DBA/1 and A/J showed an intermediate number of cells, and other strains showed relatively low numbers of Iba‐1‐immunoreactive cells (Figure 4j). Strain‐specific differences in the number of Iba‐1‐immunoreactive microglia in the dentate gyrus are shown in Figure 4j. Statistical analysis was also performed between mouse strains and showed in Figure 4j.

### 3.5 Localization of Iba‐1‐immunoreactive microglia in the CA3 region

**Figure 5:**
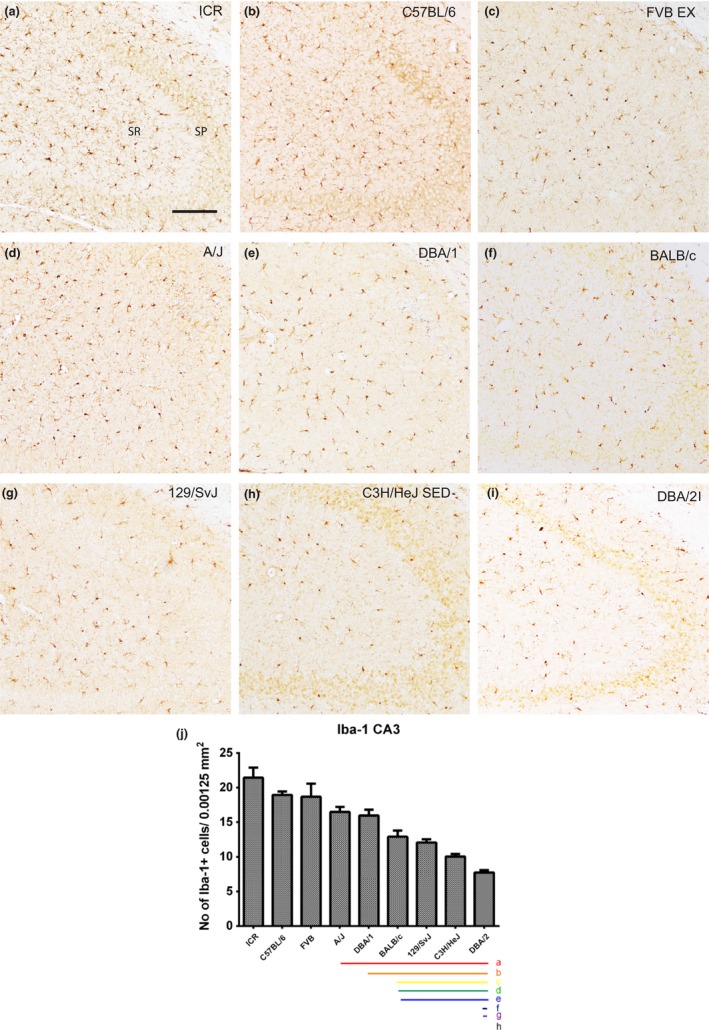
Iba‐1 immunostaining in the CA3 region and quantitative analysis of the number of Iba‐1‐immunoreactive microglia in the stratum radiatum (SR) of the CA3 region of the hippocampus. Iba‐1‐immunoreactive microglia are observed in the stratum oriens (SO) and SR of the CA3 region in all nine mouse strains; however, Iba‐1‐immunoreactive microglia show differences dependent on the mouse strain (a–i). SP, stratum pyramidal microglia. Scale bar = 100 μm. The population of Iba‐1‐immunoreactive microglia counted in nine mouse strain (j). Significant differences between the mouse strains are highlighted under the graph (j). “a” and a red bar indicate a significant difference compared to ICR C3H/HeJ; “b” and orange bar, C57BL/6; “c” and yellow bar, FVB; “d” and green bar, A/J; “e” and blue bar, DBA/1; “f” and navy bar, BALB/c; “g” and purple bar, 129/SvJ; “h” and black bar, C3H/HeJ; *p* < .05. Data are represented as mean ± *SEM*

Iba‐1‐immunoreactive microglia were also found in both the stratum oriens and radiatum of the CA3 region in all nine mouse strains. We observed significant differences in the number of Iba‐1‐immunoreactive microglia with respect to mouse strains (Figure 5a–i). The number of Iba‐1‐immunoreactive microglia was highest in the ICR (21.69 ± 1.78) and lowest in the DBA/2 (7.19 ± 0.42) strain (Figure 5j). The number of Iba‐1‐positive cells decreased progressively in the ICR, C57BL/6, FVB, A/J, DBA/1, BALB/c, 129/SvJ, C3H/HeJ, and DBA/2 strains (Figure 5j). Strain‐specific differences in the number of Iba‐1‐immunoreactive microglia in the dentate gyrus are shown in Figure 5j. Statistical analysis was also performed between mouse strains and significant showed in Figure 5j.

### 3.6 Localization of Iba‐1‐immunoreactive microglia in the polymorphic layer of the dentate gyrus

**Figure 6:**
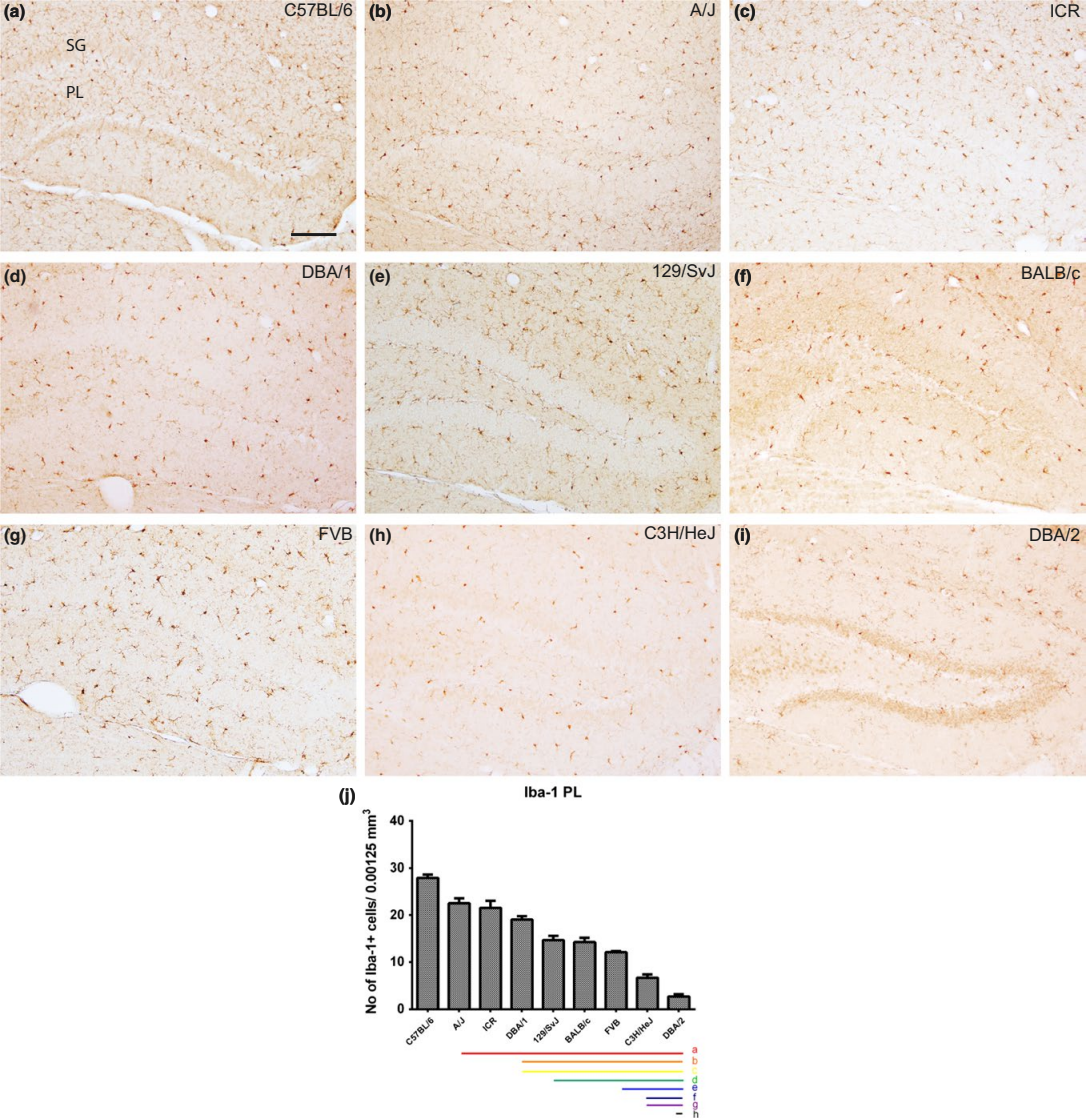
Iba‐1 immunostaining in the dentate gyrus and quantitative analysis of the number of Iba‐1‐immunoreactive microglia in the polymorphic layer (PL) of the dentate gyrus. Iba‐1‐labeled astrocytes are detected in the molecular layer (ML) and PL of the dentate gyrus in all nine mouse strains; however, Iba‐1‐immunoreactive microglia show differences dependent on mouse strain (a–i). GCL, granule cell layer. Scale bar = 100 μm. The population of Iba‐1‐immunoreactive microglia counted in nine mouse strains (j). Significant differences between mouse strains are highlighted under the graph (j). “a” and a red bar indicate a significant difference compared to C57BL/6; “b” and orange bar, A/J; “c” and yellow bar, ICR; “d” and green bar, DBA/1; “e” and blue bar, 129/SvJ; “f” and navy bar, BALB/c; “g” and purple bar, FVB; “h” and black bar, C3H/HeJ); *p* < .05. Data are represented as mean ± *SEM*

Iba‐1‐immunoreactive microglia were found in both the polymorphic layer of the dentate gyrus. The number of Iba‐1‐immunoreactive microglia was highest in the C57BL/6 (28.93 ± 0.93) and lowest in the DBA/2 (3.67 ± 0.51) strain. Progressively decreasing numbers of Iba‐1‐positive cells were seen in C57BL/6, A/J, ICR, DBA/1, 129/SvJ, BALB/c, FVB, C3H/HeJ, and DBA/2 strains (Figure 5j). The DBA/2 strain, in particular, showed a relatively very low number of Iba‐1‐immunoreactive cells in the polymorphic layer (Figure 5j). Strain‐specific differences in the number of Iba‐1‐immunoreactive microglia in the polymorphic layer of the dentate gyrus are shown in Figure 6j. Statistical analysis was also performed between mouse strains and showed in Figure 6j.

## 4 DISCUSSION

In this study, we analyzed the normal distribution and number of astrocytes and microglial cells in the hippocampus of nine mouse strains. Our data show a regional difference in the number of astrocytes and microglial cells in dorsal hippocampus from nine mouse strain. Additionally, we reported strain‐specific inherent differences in neuroglial populations within the CA1, CA3, and polymorphic layer of the hippocampus. Our key result shows a phenotypical difference in the distribution of astrocytes and microglia in the hippocampus with respect to mouse strain. This result suggests that strain‐dependent phenotypical differences can affect hippocampal‐related functions.

In the present study, we found strain‐dependent differences in the number of GFAP‐immunoreactive astrocytes in the hippocampus; C3H mice showed the largest population of GFAP‐immunoreactive astrocytes in the stratum radiatum of the CA1 and CA3 regions, and DBA/1 showed the largest population of GFAP‐immunoreactive astrocytes in the polymorphic layer of the dentate gyrus. We observed the lowest population of GFAP‐immunoreactive astrocytes in the CA1 and polymorphic layer of 129/SvJ mice and the CA3 region of FVB mice. In addition, the number of Iba‐1‐immunoreactive microglia was highest in the CA1 and CA3 regions of ICR mice and polymorphic layer of C57BL/6 mice. We observed the lowest population of Iba‐1‐immunoreactive microglial cells in the CA1 region of FVB mice and the CA3 and polymorphic layer of DBA/2 mice. Each mouse strain has unique characteristics in terms of coat color, eye color, body weight, and organ weight. C57BL/6, DBA/2, 129/SvJ, and some other mouse strains have an auditory deficiency (Zheng, Johnson, & Erway, 1999), C57BL/6 mice have a deficiency that affects melatonin synthesis in the pineal gland (Ebihara, Marks, Hudson, & Menaker, 1986), and C3H mice have a mutation that results in retinal degeneration (Chang et al., 2002). Behavioral phenotypes are also significantly different among the mouse strains. In the open field test, the C57BL/6 strain is reported to display a high level of activity, with the DBA/2, CBA, and AKR strains displaying an intermediate level of activity (DeFries, Gervais, & Thomas, 1978). In the Morris water maze task, C57BL/6 and 129 strains have a high score, while 129Sv/J, DBA/2, and BALB/c strains have a relatively poor score. The A/J has a visual impairment that affects its score in the Morris water maze task (Owen, Logue, Rasmussen, & Wehner, 1997). Additionally, in the inflammatory response activated by cerebral ischemia, the C57BL/6 and FVB mouse strains are reported to have different immune cell compositions and functional outcomes (Intlekofer & Cotman, 2013). These differences in behavioral characteristics and tasking ability, combined with our results, can provide researchers the necessary information required to choose the appropriate inbred mouse strain during the design of experiments.

We hypothesized that genes inherent to the backgrounds of these strains would influence the phenotype of the astrocyte and microglial population found in these strains. Our data show a significant difference in the number of astrocytes and microglia with respect to mouse strain and region of the hippocampus and these differences can affect the phenotype observed during a pathological status and in a neurodegenerative gene mutation model. When choosing mouse strains for mutation models of neurodegenerative disease, our data provide information that enables researchers to choose the appropriate inbred mouse strain and allows them to correlate neuroglial reactions in a neurodegenerative model with mutated genes and pathological progress.

Choosing inbred mouse strain for neurodegenerative or stroke model, many studies choose standard C57BL/6 mouse strain of jubilee outbred mouse for ischemia. Common neurological study is needed for essential and wide knowledge of origin phenotype in each inbred mouse strain, and difference of phenotype in inbred mouse may contribute for understanding of strain‐dependent genetic influence on immune system and right approach for suitable animal disease model. In a previous study about strain‐dependent difference in long‐term potentiation (LTP) and strain‐dependent memory, there are various LTP formation and spatial memory ability (Nguyen, Abel, Kandel, & Bourtchouladze, 2000), and some inbred mice have been shown to be smarter than others (Nguyen et al., 2000). Our result shows a strain‐dependent change in the number of astrocytes and microglia and demonstrates that exercise increases the number of astrocytes in only the CA1 region in all nine mouse strains. Knowledge of the function of each cell type and their interaction with each other is necessary for choosing the correct inbred mouse strain. However, the relationships between the genetic differences in each mouse strains and the basal population of astrocytes and microglia are not fully understood and remain largely undiscovered. This creates a necessity to study the strain‐dependent genetic differences in astrocyte and microglial cell populations. Additionally, there is a need for a greater understanding of how these strain‐dependent genetic differences influence astrocytes and microglial cells, not only quantitatively but also in terms of intracellular changes and mechanisms regulating these changes. Information about the differences in the phenotype of astrocytes and microglia found in the different inbred strains would provide the necessary knowledge needed to select correct genetic targets and the appropriate mouse strain to create the ideal conditions for each experiment.

Astrocytes and microglia interact with surrounding structures and regulate adjacent cell niches. In the hippocampus, astrocytes contribute to dynamic structural changes that can regulate the presentation of dendritic spines in pyramidal cells found in the CA1 region (Haber, Zhou, & Murai, 2006). Additionally, both the astrocyte ephrin‐A3 (EphA3) signal and Eph4A receptor regulate the structural formation and excitatory synaptic connection in pyramidal cells (Murai, Nguyen, Irie, Yamaguchi, & Pasquale, 2003). Astrocyte regulation of dendritic spine formation is linked to both LTP and long‐term depression (LTD) (Murakoshi & Yasuda, 2012). Astrocytes also play a role in controlling excitatory synapse formation, increasing the number of synaptic structures and regulating pre‐ and postsynaptic function via different regulatory factors. Transforming growth factor ‐β expressing astrocytes and microglia also plays a role in eliminating synapses (Chung, Allen, & Eroglu, 2015). Microglia are also known to contribute to regulating neuronal function and plasticity. Microglial brain‐derived neurotrophic factor plays a crucial role in regulating synaptic plasticity and function, and this contributes to learning‐dependent synaptic remodeling (Parkhurst et al., 2013). Microglia expressing tumor necrosis factor ‐α contribute to enhancing synaptic efficacy by increasing α‐amino‐3‐hydroxy‐5‐methyl‐4‐isoxazolepropionic acid astrocyte and microglia receptor expression (Beattie et al., 2002). In addition, nitric oxide from microglia contributes to LTP (Zhuo, Small, Kandel, & Hawkins, 1993).

The role of astrocytes and microglia in synaptic activity under normal healthy conditions is not fully understood; however, both are definitely known to contribute to synaptic activities (Ota, Zanetti, & Hallock, 2013). Astrocyte has many mechanism for formation neural synapse formation (Ota et al., 2013). Astrocyte has many mechanism for formation neural synapse formation (Nguyen et al., 2000). Our result has limitation on the strain‐dependent potency of astrocyte and microglia, and correlation of neuroglia and with strain‐dependent LTP formation, and further studies needed to identify for correlation strain‐dependent behavior and neuroglia in hippocampus. But our result gives the clue and first step for strain‐dependent behavioral, and phenotype differences on neuroglia from nine mouse strain. This difference in the phenotypes of astrocytes and microglia from inbred mouse strains would provide important information necessary for selecting genetic targets and pharmacological and neuropathological studies for appropriate mouse strains to create the ideal conditions for each experiment.
